# Development of EMS-induced mutation population for amylose and resistant starch variation in bread wheat (*Triticum aestivum*) and identification of candidate genes responsible for amylose variation

**DOI:** 10.1186/s12870-016-0896-z

**Published:** 2016-10-06

**Authors:** Ankita Mishra, Anuradha Singh, Monica Sharma, Pankaj Kumar, Joy Roy

**Affiliations:** 1 Department of Biotechnology (DBT), National Agri-Food Biotechnology Institute (NABI), Government of India, C-127 Industrial Area Phase 8, Mohali, 160071 Punjab India; 2 Department of Biotechnology, Panjab University, Chandigarh, India

**Keywords:** *Triticum aestivum*, Ethyl methanesulfonate, Amylose, Resistant starch, Starch metabolic pathway genes, qRT-PCR

## Abstract

**Background:**

Starch is a major part of cereal grain. It comprises two glucose polymer fractions, amylose (AM) and amylopectin (AP), that make up about 25 and 75 % of total starch, respectively. The ratio of the two affects processing quality and digestibility of starch-based food products. Digestibility determines nutritional quality, as high amylose starch is considered a resistant or healthy starch (RS type 2) and is highly preferred for preventive measures against obesity and related health conditions. The topic of nutrition security is currently receiving much attention and consumer demand for food products with improved nutritional qualities has increased. In bread wheat (*Triticum aestivum* L.), variation in amylose content is narrow, hence its limited improvement. Therefore, it is necessary to produce wheat lines or populations showing wide variation in amylose/resistant starch content. In this study, a set of EMS-induced M4 mutant lines showing dynamic variation in amylose/resistant starch content were produced. Furthermore, two diverse mutant lines for amylose content were used to study quantitative expression patterns of 20 starch metabolic pathway genes and to identify candidate genes for amylose biosynthesis.

**Results:**

A population comprising 101 EMS-induced mutation lines (M4 generation) was produced in a bread wheat (*Triticum aestivum*) variety. Two methods of amylose measurement in grain starch showed variation in amylose content ranging from ~3 to 76 % in the population. The method of in vitro digestion showed variation in resistant starch content from 1 to 41 %. One-way ANOVA analysis showed significant variation (*p* < 0.05) in amylose and resistant starch content within the population. A multiple comparison test (Dunnett’s test) showed that significant variation in amylose and resistant starch content, with respect to the parent, was observed in about 89 and 38 % of the mutant lines, respectively. Expression pattern analysis of 20 starch metabolic pathway genes in two diverse mutant lines (low and high amylose mutants) showed higher expression of key genes of amylose biosynthesis (GBSSI and their isoforms) in the high amylose mutant line, in comparison to the parent. Higher expression of amylopectin biosynthesis (SBE) was observed in the low amylose mutant lines. An additional six candidate genes showed over-expression (BMY, SPA) and reduced-expression (SSIII, SBEI, SBEIII, ISA3) in the high amylose mutant line, indicating that other starch metabolic genes may also contribute to amylose biosynthesis.

**Conclusion:**

In this study a set of 101 EMS-induced mutant lines (M4 generation) showing variation in amylose and resistant starch content in seed were produced. This population serves as useful germplasm or pre-breeding material for genome-wide study and improvement of starch-based processing and nutrition quality in wheat. It is also useful for the study of the genetic and molecular basis of amylose/resistant starch variation in wheat. Furthermore, gene expression analysis of 20 starch metabolic genes in the two diverse mutant lines (low and high amylose mutants) indicates that in addition to key genes, several other genes (such as phosphorylases, isoamylases, and pullulanases) may also be involved in contributing to amylose/amylopectin biosynthesis.

**Electronic supplementary material:**

The online version of this article (doi:10.1186/s12870-016-0896-z) contains supplementary material, which is available to authorized users.

## Background

Bread wheat (*Triticum aestivum* L.) is a staple cereal crop and a major source of carbohydrates, mainly starch. Starch is a complex glucose polymer that presents in a granular form known as a starch granule. Starch granules comprise two distinct glucose polymers - amylose (mainly a linear polymer) and amylopectin (a highly branched polymer) – consisting of about 25 % (amylose) and 75 % (amylopectin) of total starch, respectively. Their composition affects processing, cooking, organoleptic, and nutritional quality of end-use food products. Starch has wide applications in food industries where it is modified by chemical treatment as per requirement. Amylose or amylopectin fractions, however, have been altered in plants *per se* through extensive breeding approaches as well as using advanced functional genomics tools to improve processing and nutritional quality. For example, partial waxy wheats have been developed by decreasing waxy proteins (GBSSI proteins) to create low amylose wheat which is used in the production of good-quality noodles. [1–5]. High amylose wheats have been developed using advanced functional genomics tools, as well as EMS treatments and breeding approaches [6–14]. Amylose has been increased to make ‘Type 2 resistant starch’ (‘RS 2’) for improving nutritional quality. It is found that high amylose starch (HA) is digested slower than normal starch in the stomach and small intestine, similar to dietary fiber [13, 15]. It has a low glycemic index and, therefore, it can be used to make low glycemic index food products for people with obesity or diabetes. Further, high amylose starch is fermented in the lower intestine to release small chain fatty acids (SCFAs), which provide additional health benefits to colon health and brain tissues. The detailed account of the functionality and application of low and high amylose wheat starches is given elsewhere [16].

Amylose is predominately a linear glucan polymer chain of a few hundred to a few thousand glucose units linked by α-1,4-linkages, whereas amylopectin is a highly branched glucan polymer chain of many thousands of glucose units with α-1,4 and α-1,6 linkages [17]. Starch is biosynthesized within the amyloplasts from glucose-1-phosphate. Starch biosynthesis is initiated by ADP-glucose pyrophosphorylase (AGPase) from glucose-1-phosphate in seed amyloplasts and further by a series of several classes of enzymes whose isoforms are involved in the biosynthesis of amylose and amylopectin [18]. Amylose is biosynthesized by granule-bound starch synthase (GBSS) while amylopectin is biosynthesized by the coordinated actions of soluble starch synthase (SS), starch branching enzyme (SBE), and starch debranching enzyme (DBEs) [19]. Starch metabolic pathway genes responsible for the modulation of the amylose-amylopectin ratio have been identified either through extensive breeding approaches [1–3] or through advance biotechnological approaches, including T-DNA or transposon insertion [14, 20] and RNAi [13].

Chemical agents have been used to produce phenotypic variation. Among them, ethyl methanesulfonate (EMS) has been widely used in crops [21]. It is an alkylating agent directly affecting DNA by alkylating guanine (G) bases, causing mispairing with thiamine (T) instead of cytosine (C), resulting in a transition from G/C to A/T [22]. This is preferable to other biotechnological approaches as it produces a large spectrum of mutations and allows multiple alleles of a specific gene in a small population. EMS-induced mutagenesis has been widely used to produce novel allelic variation in genes which are involved in starch biosynthesis. Partial null-waxy and complete waxy phenotypes were produced by targeting the loci of the gene encoding GBSSI in wheat [5, 23]. In addition, other starch metabolic genes such as SBEIIa, SBEIIb and SSIIa were also targeted for development of low or high amylose starch in wheat [6–8, 10, 12].

Amylose possesses a unique biochemical property, as it forms a deep blue color when exposed to iodine in solution. Its linear glucan chains form briefly and coil around iodine molecules, creating a non-polar environment, which changes the refractive index and results in a deep blue color [24]. It is believed that estimation of amylose content by iodine binding may be an overestimate due to it binding also with long branches of amylopectin, if present. Therefore amylose content, as estimated by the traditional iodine reaction, is sometimes designated as “apparent amylose” or “amylose-equivalent”. However, using a calibration curve and standards of known amylose content of related crop species, the overestimation can be minimized [25]. Identification of genes/QTL using natural variations in a heterogeneous population is a challenging task [26]. It is highly advocated to use near isogenic lines and/or functional genomic tools such as RNAi [13] or genome editing [27]. Both approaches have been successfully used in wheat. A set of mutant lines in the same genetic background showing the dynamic range of variation in amylose content are required for genome-wide analysis to understand amylose or amylopectin biosynthesis. In this study, a set of EMS treated mutant lines showing continuous variation in amylose and resistant starch content have been developed in a bread wheat variety. Further, one high amylose mutant line and one low amylose mutant line were used to study quantitative gene expression patterns of 20 starch metabolic pathway genes during seed development.

## Results and discussion

### Advance generation of EMS-induced population in wheat

The bread wheat variety, ‘C 306’, used in this study was released in 1965 in India (pedigree: <RGN/CSK3//2*C591/3/C217/N14// C281>). EMS (0.2 %) treatment of ~5000 seeds (M0) of the parent bread wheat variety ‘C 306’ produced ~2400 M1 plants with a germination rate of ~50 %. The M1 plants were self-pollinated and individual spikes of primary tillers were collected to produce ~1400 M2 seeds. These were sown and generated 1035 M3 seeds. The majority of M3 plants were morphologically homogeneous, resembling the parental type, and thus used for further analysis. Mutant lines differing in height, leaf color, and morphology were not used. Different concentrations of EMS (0.2 to 1.0 %) have been previously used to create mutant populations in wheat [12, 23, 28–30]. The EMS treated lines were used to identify mutations in candidate genes of interest in diploid [29], tetraploid [12, 23, 30], and hexaploid wheat [12, 31]. EMS concentrations used in this study were able to produce variation in amylose content (described later).

### Evaluation of amylose variation in mutant lines

**Fig. 1:**
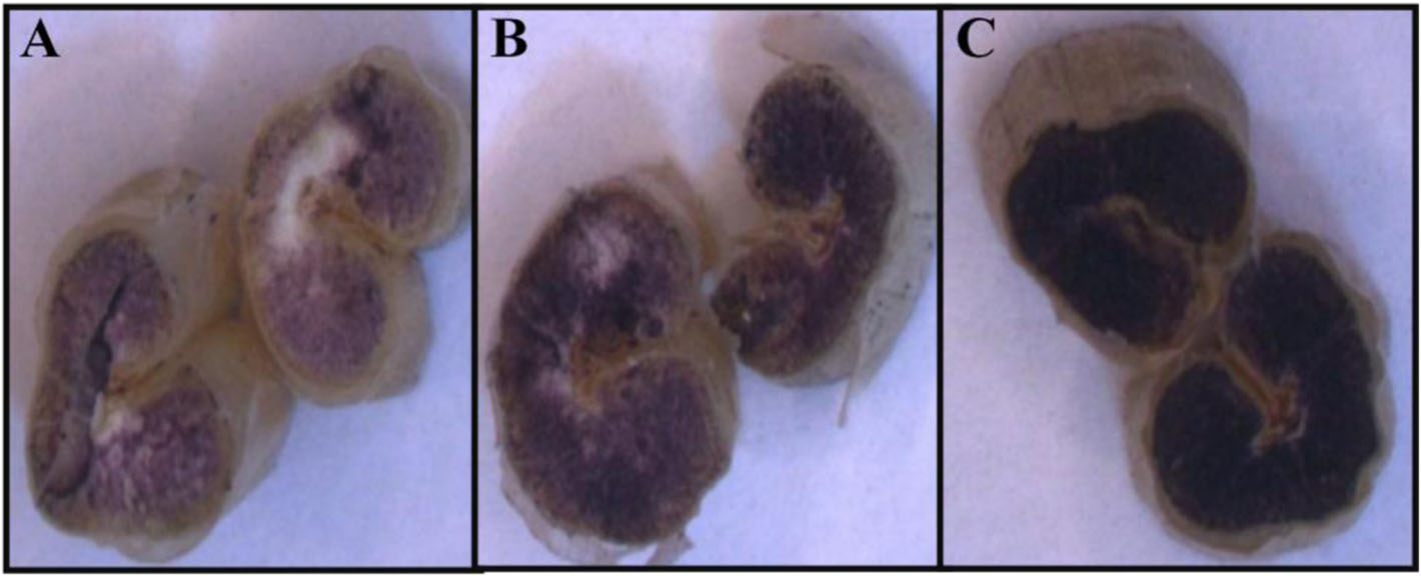
Blue color intensity on half-seeds of two EMS treated mutant lines and the parent variety varying in amylose content. Low (**a**), intermediate (**b**), and high (**c**) color intensity were observed in seeds of the low amylose mutant line (Amylose content – 6 %), the parent variety (Amylose content – 26 %), and the high amylose mutant line (Amylose content – 64 %), respectively

**Fig. 2:**
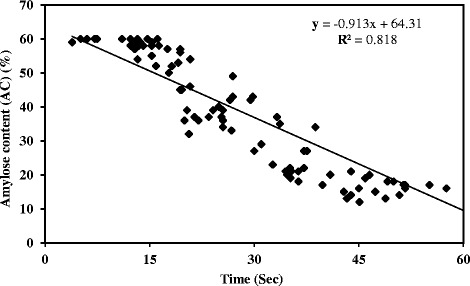
Regression analysis of amylose content (%) on time taken (sec) to develop blue color in the 101 EMS treated M4 mutant lines. The amylose content was measured in starch extracted from grains of the mutant lines and time taken (sec) to develop blue color was recorded for the half-seeds of the mutant lines soaked in Iodine-Potassium Iodide (I_2_– KI) solution

A traditional Iodine-Potassium Iodide (I_2_-KI) solution showed variation in blue color on half-seeds of 1035 M3 mutant lines (Fig. 1). The lines were subjectively divided into three groups on the basis of blue color intensity. The first group comprised 61 lines that did not develop color, indicating low amylose content. The second group comprised 886 lines that developed light blue color intensity, indicating intermediate amylose content. The third group comprised 88 lines that developed a high intensity blue color indicating high amylose content (Additional file 1). Further, we observed variation in the time taken to develop blue color. The data on the time taken to develop blue color is provided in Additional file 1. A subset of 101 mutant lines, taken from the three groups of 1035 M3 mutant lines, was selected on the basis of color intensity and time taken to develop color. Measurements of amylose/resistant starch content were taken for this subset. Further regression analysis between the time taken to develop blue color and the measured amylose content in the 101 mutant lines (described later) showed a significant negative correlation value (*r* = −0.904, *p* ≤ 0.05), indicating a negative relationship between time taken to develop blue color and increased AC (Fig. 2), which is in agreement with previous results [32]. Amylose content prediction by single-seed or half-seed has been well established for a variety of cereals such as wheat [33], rice [34], and barley [35]. The data on intensity and time taken to develop blue color on half-seed using a five-times diluted I_2_-KI standard solution would be useful for screening large populations for low, intermediate, and high amylose content predictions in wheat breeding programs.

Amylose measurements in the starch of 101 mutant lines (M4 generation) obtained by using two methods - traditional I_2_-KI and Con A methods - showed variation in amylose content ranging from ~3 (‘TAC 358’) to 76 % (‘TAC 399’) (Table 1; Additional file 2). While both methods showed similar amylose content in measured lines, there were a few exceptions. One-way ANOVA analysis showed no significant variation (*p* = 0.99) between the amylose content data from the two methods. Furthermore, the data from two biological replicates showed similar amylose content to the 101 mutant lines. One-way ANOVA analysis showed no significant variation in amylose content of the lines in the two biological replications (*p* = 0.99). The similarity and strong correlation between traditional iodine binding and Megazyme’s Con A methods of amylose measurement was reported earlier [25]. The two methods of amylose measurement and the biological replicates indicated that amylose content in these mutant lines is consistent and stable in the M4 generation. The ANOVA analysis showed significant differences (*p* < 0.05) among the 101 mutant lines for amylose content. A multiple comparison test (Dunnett’s test) of mean data for each mutant line, with respect to the parent variety ‘C 306’, showed significant differences in 90 mutant lines. This indicates that the majority of the mutant lines (~89 %) showed significant variation in amylose content from the parent variety.

**Table 1:** Evaluation of amylose content, resistant starch content, and thousand kernel weight (TKW) in the 101 EMS treated M4 mutant lines

Mutant lines	Amylose Content (AC)	Resistant Starch (RS)	TKW	Amylose Content (AC)	Resistant Starch (RS)	TKW
	Biological replication # 1			Biological replication # 2		
‘C 306’ (parent)	26.2 ± 0.4	00.8 ± 0.1	40.6 ± 0.14	26.6 ± 0.0	00.5 ± 0.5	40.3 ± 0.4
HAM (66 %)	65.5 ± 0.4	36.8 ± 0.0	-	65.5 ± 0.4	36.9 ± 1.1	-
TAC 6	06.8 ± 0.0	00.2 ± 0.3	46.0 ± 0.21	06.6 ± 0.1	00.8 ± 1.2	45.9 ± 0.1
TAC 28	73.2 ± 0.4	39.6 ± 1.2	48.1 ± 0.57	73.1 ± 0.3	45.3 ± 0.1	48.0 ± 0.4
TAC 35	68.7 ± 0.4	30.2 ± 0.2	41.2 ± 1.06	68.5 ± 0.3	28.4 ± 0.5	40.9 ± 0.6
TAC 51	67.0 ± 0.2	30.4 ± 1.1	39.2 ± 0.42	67.1 ± 0.2	30.7 ± 0.4	39.2 ± 0.5
TAC 71	68.7 ± 0.1	32.4 ± 0.1	43.2 ± 0.42	68.6 ± 0.4	34.9 ± 0.8	42.9 ± 0.1
TAC 74	69.2 ± 0.5	35.4 ± 0.7	45.7 ± 0.35	69.0 ± 0.3	37.4 ± 2.0	51.4 ± 8.3
TAC 75	64.4 ± 0.4	37.8 ± 1.1	49.0 ± 0.28	64.2 ± 0.5	35.8 ± 0.2	49.2 ± 0.5
TAC 104	04.4 ± 0.6	00.0 ± 0.0	47.2 ± 0.42	04.6 ± 0.1	00.0 ± 0.0	47.4 ± 0.7
TAC 137	18.5 ± 0.3	00.7 ± 0.0	45.8 ± 0.28	18.6 ± 0.1	01.2 ± 0.7	45.8 ± 0.3
TAC 163	16.3 ± 0.5	00.3 ± 0.0	42.8 ± 0.57	16.1 ± 0.1	00.5 ± 0.0	43.2 ± 1.0
TAC 176	13.8 ± 0.2	00.1 ± 1.1	44.0 ± 0.42	13.6 ± 0.2	02.1 ± 0.2	43.9 ± 0.2
TAC 197	24.8 ± 0.3	00.4 ± 0.3	44.8 ± 0.28	25.1 ± 0.2	00.4 ± 0.3	45.2 ± 1.0
TAC 237	07.7 ± 0.3	00.1 ± 0.7	40.7 ± 0.78	07.5 ± 0.2	02.2 ± 0.1	40.6 ± 0.5
TAC 243	43.6 ± 0.3	10.0 ± 0.9	47.6 ± 0.42	43.7 ± 0.1	10.0 ± 0.9	47.9 ± 0.9
TAC 273	25.9 ± 0.1	00.9 ± 0.8	47.7 ± 0.42	25.7 ± 0.1	02.2 ± 0.1	48.0 ± 0.8
TAC 287	35.7 ± 0.2	01.2 ± 1.2	43.6 ± 0.49	35.5 ± 0.2	00.4 ± 0.1	43.9 ± 0.9
TAC 288	11.6 ± 0.5	00.3 ± 0.5	41.7 ± 0.35	11.8 ± 0.2	00.3 ± 0.5	42.2 ± 0.9
TAC 308	37.4 ± 0.3	02.0 ± 1.7	45.9 ± 0.57	36.9 ± 0.3	04.5 ± 0.3	46.2 ± 1.1
TAC 354	20.4 ± 0.7	00.5 ± 0.2	32.8 ± 0.35	20.2 ± 0.1	01.0 ± 0.4	34.1 ± 2.1
TAC 358	02.6 ± 0.5	00.1 ± 0.2	42.2 ± 0.99	02.9 ± 0.3	00.3 ± 0.0	42.1 ± 0.9
TAC 360	46.4 ± 0.2	12.1 ± 1.4	43.0 ± 0.78	46.7 ± 0.3	12.3 ± 1.5	42.7 ± 0.4
TAC 362	35.9 ± 0.6	02.6 ± 0.7	41.7 ± 0.71	36.2 ± 0.4	03.6 ± 0.6	41.2 ± 0.3
TAC 369	39.3 ± 0.5	07.6 ± 0.7	45.4 ± 0.35	39.1 ± 0.2	10.0 ± 1.3	45.6 ± 0.5
TAC 374	14.6 ± 0.6	00.0 ± 0.1	46.7 ± 0.49	14.6 ± 0.1	00.0 ± 0.1	47.5 ± 0.9
TAC 380	26.3 ± 0.3	01.3 ± 1.3	41.0 ± 0.21	26.0 ± 0.3	02.8 ± 0.7	41.0 ± 0.1
TAC 381	29.2 ± 0.2	01.7 ± 1.1	45.2 ± 0.07	29.5 ± 0.0	03.2 ± 0.9	45.8 ± 0.8
TAC 399	75.7 ± 0.4	41.3 ± 0.1	39.2 ± 0.49	76.0 ± 0.4	42.6 ± 0.1	39.5 ± 0.8
TAC 404	46.8 ± 0.5	14.7 ± 1.4	46.0 ± 0.71	46.4 ± 0.1	16.2 ± 0.6	46.2 ± 0.9
TAC 418	35.3 ± 0.5	01.5 ± 0.1	48.2 ± 0.07	35.0 ± 0.1	01.5 ± 0.6	48.7 ± 0.7
TAC 419	32.0 ± 0.3	01.2 ± 0.0	46.5 ± 0.49	32.5 ± 0.1	01.2 ± 0.0	46.4 ± 0.2
TAC 421	36.4 ± 0.2	01.5 ± 0.3	32.9 ± 0.57	36.3 ± 0.0	02.0 ± 1.0	32.7 ± 0.4
TAC 423	20.0 ± 0.2	00.0 ± 1.1	46.7 ± 0.28	19.8 ± 0.2	01.5 ± 1.9	46.9 ± 0.6
TAC 428	43.4 ± 0.2	12.7 ± 0.8	37.6 ± 0.64	43.9 ± 0.1	07.7 ± 0.8	37.6 ± 0.6
TAC 437	51.5 ± 0.3	14.4 ± 1.2	43.9 ± 0.35	51.5 ± 0.1	14.5 ± 1.9	44.2 ± 0.7
TAC 457	34.9 ± 0.2	01.3 ± 0.6	41.9 ± 0.35	34.4 ± 0.0	02.8 ± 1.5	41.8 ± 0.3
TAC 477	35.5 ± 0.3	02.3 ± 1.4	45.6 ± 0.64	35.6 ± 0.1	06.3 ± 1.3	45.4 ± 0.3
TAC 536	16.4 ± 0.1	00.3 ± 0.3	51.2 ± 0.35	16.3 ± 0.0	02.8 ± 1.7	50.7 ± 1.0
TAC 539	16.1 ± 0.2	00.0 ± 0.1	48.1 ± 0.28	16.1 ± 0.0	00.5 ± 0.4	49.0 ± 0.9
TAC 560	19.7 ± 0.0	00.7 ± 0.0	46.2 ± 0.35	19.6 ± 0.1	01.2 ± 0.7	46.8 ± 0.6
TAC 587	13.1 ± 0.3	00.5 ± 0.2	41.1 ± 0.35	13.2 ± 0.0	01.5 ± 1.6	42.0 ± 0.8
TAC 606	43.2 ± 0.0	10.2 ± 0.1	48.1 ± 0.14	42.5 ± 0.1	09.7 ± 0.5	48.5 ± 0.7
TAC 622	42.7 ± 0.2	09.7 ± 0.4	39.1 ± 0.14	40.8 ± 0.0	00.2 ± 0.2	40.6 ± 2.0
TAC 623	20.9 ± 0.4	00.2 ± 0.1	46.5 ± 0.57	20.8 ± 0.0	08.4 ± 0.5	46.4 ± 0.6
TAC 636	42.0 ± 0.2	14.7 ± 0.2	42.6 ± 0.42	42.6 ± 0.0	13.7 ± 1.1	43.3 ± 0.7
TAC 662	44.4 ± 0.5	15.6 ± 0.9	52.1 ± 0.21	44.8 ± 0.0	02.1 ± 0.2	52.6 ± 0.5
TAC 681	26.9 ± 0.7	00.8 ± 1.5	48.8 ± 0.14	26.8 ± 0.1	09.6 ± 1.0	48.9 ± 0.2
TAC 696	46.8 ± 0.3	15.4 ± 0.1	39.2 ± 0.35	43.7 ± 0.1	06.4 ± 1.6	39.6 ± 0.9
TAC 703	15.2 ± 0.2	00.4 ± 0.0	49.4 ± 0.28	17.8 ± 0.1	00.2 ± 0.1	49.6 ± 0.6
TAC 14	63.4 ± 0.6	23.4 ± 1.1	41.1 ± 0.21	63.4 ± 0.0	22.4 ± 0.2	45.7 ± 0.7
TAC 708	23.8 ± 0.2	00.5 ± 0.3	45.4 ± 0.28	23.7 ± 0.1	01.2 ± 0.3	47.8 ± 1.0
TAC 711	17.9 ± 0.2	00.1 ± 0.2	47.5 ± 0.57	17.8 ± 0.1	00.1 ± 0.2	46.0 ± 1.0
TAC 713	13.4 ± 0.0	00.3 ± 0.7	43.3 ± 0.00	13.4 ± 0.0	02.0 ± 0.6	43.7 ± 0.5
TAC 730	11.2 ± 0.3	00.2 ± 0.1	47.7 ± 0.21	11.2 ± 0.0	02.3 ± 2.2	46.8 ± 1.5
TAC 737	13.0 ± 0.5	00.2 ± 0.3	43.4 ± 0.14	12.9 ± 0.1	00.4 ± 0.1	43.7 ± 0.4
TAC 741	18.5 ± 0.4	00.3 ± 0.0	46.7 ± 0.21	18.3 ± 0.3	00.8 ± 0.7	46.5 ± 0.2
TAC 747	24.5 ± 0.3	00.5 ± 0.5	47.2 ± 0.14	24.5 ± 0.1	00.0 ± 0.1	47.6 ± 0.5
TAC 748	25.7 ± 0.4	00.1 ± 0.2	44.5 ± 0.07	25.4 ± 0.3	00.1 ± 0.2	45.0 ± 0.8
TAC 765	15.9 ± 0.3	00.3 ± 0.9	40.5 ± 0.49	15.7 ± 0.2	01.6 ± 0.9	40.4 ± 0.2
TAC 766	19.3 ± 0.3	00.5 ± 0.7	49.6 ± 0.49	19.5 ± 0.1	00.5 ± 0.7	49.7 ± 0.6
TAC 781	29.4 ± 0.0	00.7 ± 2.9	42.4 ± 0.21	29.4 ± 0.0	03.2 ± 0.8	42.7 ± 0.5
TAC 790	40.9 ± 0.5	09.7 ± 0.5	41.6 ± 0.35	40.7 ± 0.2	09.7 ± 0.5	41.7 ± 0.4
TAC 810	19.1 ± 0.0	00.4 ± 0.3	46.4 ± 0.57	19.0 ± 0.0	00.9 ± 0.3	47.2 ± 0.6
TAC 824	49.1 ± 0.4	14.2 ± 0.2	38.1 ± 0.49	49.2 ± 0.1	14.7 ± 0.9	38.9 ± 1.6
TAC 831	33.2 ± 0.2	01.7 ± 0.0	48.7 ± 0.28	33.3 ± 0.1	05.2 ± 0.7	48.9 ± 0.6
TAC 846	07.1 ± 0.2	00.3 ± 0.5	50.6 ± 0.57	07.1 ± 0.1	00.3 ± 0.5	50.6 ± 0.6
TAC 869	26.9 ± 0.2	00.9 ± 0.4	42.7 ± 0.42	26.8 ± 0.1	01.9 ± 0.9	43.0 ± 0.8
TAC 880	12.4 ± 0.2	00.2 ± 0.1	45.4 ± 0.64	12.5 ± 0.1	00.0 ± 0.0	45.5 ± 0.7
TAC 902	20.4 ± 0.2	00.5 ± 0 .2	45.1 ± 0.21	20.5 ± 0.1	01.0 ± 0.4	46.2 ± 1.4
TAC 903	29.3 ± 0.2	01.0 ± 0.2	43.7 ± 0.64	29.5 ± 0.1	01.4 ± 0.8	43.7 ± 0.5
TAC 914	17.5 ± 0.3	00.2 ± 0.1	40.9 ± 0.14	17.7 ± 0.2	00.2 ± 0.1	41.2 ± 0.5
TAC 917	27.1 ± 0.3	00.5 ± 1.8	48.2 ± 0.57	26.9 ± 0.2	01.5 ± 1.8	47.9 ± 0.2
TAC 942	33.8 ± 0.1	01.2 ± 1.4	45.1 ± 0.14	33.8 ± 0.1	02.2 ± 0.0	45.6 ± 0.9
TAC 947	50.8 ± 0.4	12.0 ± 0.6	42.1 ± 0.14	50.6 ± 0.1	13.0 ± 0.7	42.9 ± 0.9
TAC 955	26.5 ± 0.2	00.5 ± 0.2	40.0 ± 0.42	26.6 ± 0.1	01.5 ± 1.6	39.9 ± 0.3
TAC 975	55.1 ± 0.2	19.7 ± 14.	47.7 ± 0.35	55.2 ± 0.0	20.2 ± 8.4	48.0 ± 0.7
TAC 981	11.6 ± 0.3	00.1 ± 0.9	45.5 ± 0.49	11.4 ± 0.2	02.1 ± 0.2	45.6 ± 0.5
TAC 989	32.0 ± 0.5	01.3 ± 1.4	43.4 ± 0.35	31.8 ± 0.2	02.8 ± 0.7	43.9 ± 0.9
TAC 990	25.5 ± 0.1	00.8 ± 1.7	35.3 ± 0.35	25.4 ± 0.1	02.8 ± 0.3	37.3 ± 2.4
TAC 1024	51.9 ± 0.3	12.6 ± 2.3	29.9 ± 0.85	51.2 ± 0.2	11.6 ± 0.9	32.1 ± 4.1
TAC 1025	11.6 ± 0.5	00.2 ± 0.9	49.0 ± 0.07	11.8 ± 0.3	01.4 ± 0.2	49.7 ± 0.9
TAC 1026	12.6 ± 0.2	00.0 ± 0.1	40.7 ± 0.42	12.5 ± 0.1	00.0 ± 0.1	40.8 ± 0.5
TAC 1046	16.1 ± 0.4	00.2 ± 0.0	44.9 ± 0.92	15.8 ± 0.3	01.2 ± 1.3	44.9 ± 0.8
TAC 1054	14.6 ± 0.3	00.3 ± 0.0	42.9 ± 0.99	14.4 ± 0.2	01.4 ± 0.7	42.9 ± 1.0
TAC 1068	35.9 ± 0.2	02.1 ± 0.0	37.2 ± 0.85	21.1 ± 0.3	02.1 ± 0.0	37.8 ± 1.7
TAC 1072	37.3 ± 0.1	02.7 ± 0.2	37.4 ± 0.64	40.0 ± 0.0	03.7 ± 0.2	38.4 ± 0.7
TAC 1075	21.2 ± 0.2	00.6 ± 0.5	39.0 ± 0.71	46.3 ± 0.2	00.8 ± 0.5	40.0 ± 0.7
TAC 1081	12.8 ± 0.2	00.0 ± 2.9	45.6 ± 0.49	46.7 ± 0.2	00.8 ± 1.4	45.8 ± 0.7
TAC 1090	57.1 ± 0.2	19.9 ± 3.4	32.5 ± 0.71	47.6 ± 0.1	16.9 ± 0.7	32.0 ± 0.0
TAC 1151	52.6 ± 0.5	14.8 ± 0.6	46.8 ± 0.42	7.3 ± 0.0	15.8 ± 0.6	47.0 ± 0.7
TAC 1168	49.2 ± 0.0	21.0 ± 2.0	44.1 ± 0.64	50.0 ± 0.1	18.5 ± 1.4	44.1 ± 0.5
TAC 1171	63.4 ± 0.6	37.6 ± 3.3	46.2 ± 0.49	36.0 ± 0.1	06.1 ± 1.1	46.4 ± 0.8
TAC 1193	73.2 ± 0.4	40.9 ± 1.5	41.1 ± 0.21	73.4 ± 0.1	42.4 ± 0.8	41.8 ± 0.7
TAC 1194	68.7 ± 0.4	36.5 ± 0.2	47.4 ± 0.35	21.0 ± 0.1	35.0 ± 0.4	48.2 ± 0.6
TAC 1201	67.0 ± 0.2	36.6 ± 0.1	40.9 ± 0.57	12.9 ± 0.1	34.6 ± 0.1	40.8 ± 0.3
TAC 1202	68.7 ± 0.1	36.2 ± 0.5	39.2 ± 0.49	57.2 ± 0.1	33.2 ± 1.9	38.9 ± 0.1
TAC 1207	35.7 ± 0.2	02.6 ± 1.7	42.7 ± 0.28	52.4 ± 0.2	03.6 ± 1.7	43.9 ± 1.5
TAC 364	40.2 ± 0.5	03.8 ± 1.8	60.0 ± 0.14	40.2 ± 0.1	03.3 ± 1.1	58.0 ± 2.8
TAC 172	30.1 ± 0.3	01.5 ± 1.6	60.0 ± 1.20	30.0 ± 0.0	02.0 ± 0.9	59.5 ± 2.1
TAC 988	23.8 ± 0.2	00.6 ± 0.4	62.0 ± 0.99	23.7 ± 0.1	01.1 ± 1.1	61.4 ± 1.9
TAC 1105	24.7 ± 1.0	00.6 ± 0.1	50.6 ± 0.21	24.7 ± 0.0	01.1 ± 0.8	50.9 ± 0.1

Amylose content was measured by Concanavalin A (Con A) method in seed starch. Resistant starch content was measured through a modified protocol of Megazyme. Thousand kernel weight (grams) was recorded on randomly selected seeds

Out of 101 mutant lines, 48 showed >30 % AC, indicating high amylose mutant lines and 17 lines showed <15 % AC, indicating low amylose mutant lines. Within the high amylose lines, three lines showed >70 % AC, ten showed 60–70 % AC, and five showed 50–60 % AC. In the low amylose lines, two lines showed <5 % AC, three showed 5–10 % AC, and 12 showed 10–15 % AC. Individual high amylose lines with 70–85 % AC [11, 13] have been developed in wheat. Other high amylose lines with 30–60 % AC have been reported in wheat including diploid, tetraploid, and hexaploid species [6–8, 36]. Similarly, individual low amylose lines (i.e. waxy and partial waxy wheat lines) have been developed [2, 37]. In this study, a wide range of high amylose lines with 30 to 76 % AC have been developed in the same genetic background in wheat using EMS. Therefore, these lines would be useful for genome-wide analysis of the genetic and molecular basis of amylose variation in wheat.

### Measurement of resistant starch in mutant lines

Resistant starch measurements showed a variation from about 1 to 41 % in the mutant lines (Table 1). Twelve mutant lines showed very high resistant starch content (>30 %). Sixteen mutant lines showed 5 to 30 % resistant starch content. ANOVA analysis showed significant differences (*p* < 0.05) in resistant starch (RS) content of the 101 mutant lines and no significant differences were observed between the biological replicates. A multiple comparison test (Dunnett’s test) of mean data for each mutant line, with respect to the parent variety ‘C 306’, showed significant differences in 38 mutant lines. This indicates that significant variation in resistant starch content, with respect to the parent, was observed in about ~38 % of the 101 mutant lines, whereas variation in amylose content was observed in ~89 % of the 101 mutant lines. The amylose content of the 38 mutant lines was between 42–76 %. The resistant starch content in the high amylose lines reported in the published literatures was between ~1 to 14 % [6, 9, 11]. In this study, 18 mutant lines showed >15 % resistant starch content. These lines would be useful for genome-wide analysis of the genetic and molecular basis of resistant starch variation as well as the improvement of nutritional quality in wheat.

### Evaluation of thousand-kernel weight in the mutant lines

Thousand-kernel weight (TKW) of the 101 mutant lines ranged from about 32 g (‘TAC 1024’) to 62 g (‘TAC 988’) and that of the parent variety, ‘C 306’, was about 40 g (Table 1). A multiple comparison test (Dunnett’s test) of mean data for each mutant line with respect to the parent variety, ‘C 306’, showed significant differences in 84 mutant lines. This indicates that the majority of these mutant lines have better grain weights than that of the parent variety. Statistical correlation analysis (Pearson’s correlation) of TKW with amylose and resistant starch content of the mutant lines were −0.204 (r) and −0.102 (r), respectively, indicating poor negative correlations. The TKW correlation analysis of the mutant lines with >30 % amylose content and >5 % RS showed −0.124 (r) and 0.0054 (r), respectively, still indicating poor correlation. Correlation analysis of amylose content of low amylose lines, i.e. partial waxy mutant lines, (<15 % AC) with their TKW showed slightly strong negative correlations (*r* = −0.387). Observations reported by [37] show a lower or similar TKW of EMS-treated waxy bread wheat lines to those of the wild type. In this study, most of the high amylose mutant lines that have better grain weights than that of the parent variety would be useful in wheat improvement breeding for high amylose.

### Quantitative expression analysis of starch metabolic pathway genes in high and low amylose mutant lines

**Fig. 3:**
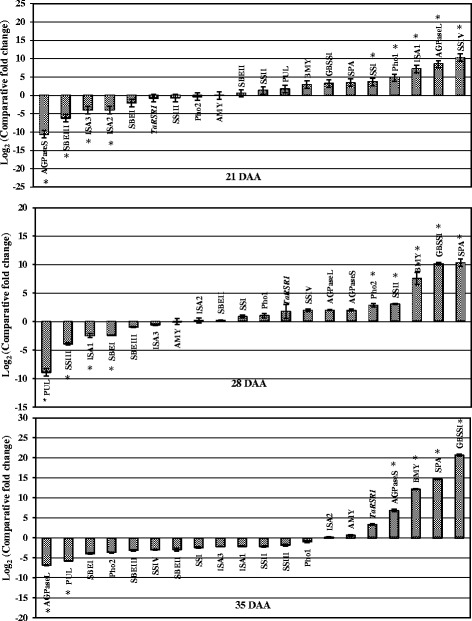
Real-time quantitative expression data (Log_2_ of fold change) of 20 starch metabolic genes during seed development in the high amylose (amylose content - 64 %) mutant line, ‘TAC 75’, in comparison to the parent variety, ‘C 306’ (amylose content – 26 %). The seed development stages were 21, 28, and 35 days after anthesis (DAA). All the data are represented as mean ± SD from two biological and three technical replicates. The symbol ‘*’ indicates significant difference at *P* < 0.05

**Fig. 4:**
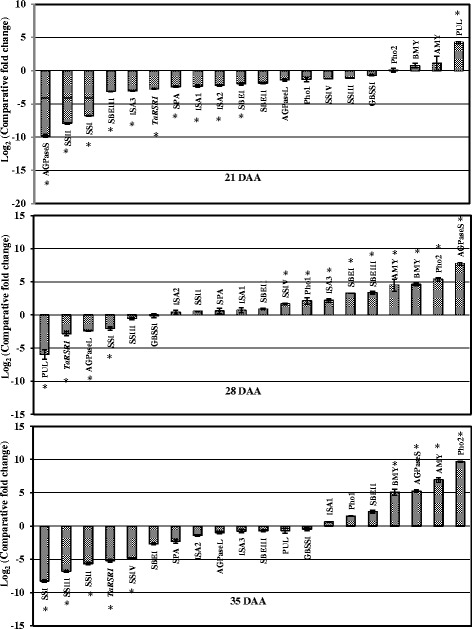
Real-time quantitative expression data (Log_2_ of fold change) of 20 starch metabolic genes during seed development in the low amylose (amylose content– 6.8 %) mutant line, ‘TAC 6’, in comparison to the parent variety, ‘C 306’ (amylose content - 26 %). The seed development stages were 21, 28, and 35 days after anthesis (DAA). All the data are represented as mean ± SD from two biological and three technical replicates. The symbol ‘*’ indicates significant difference at *P* < 0.05

In order to study the expression patterns of 20 starch metabolic pathway genes, including the genes responsible for amylose and amylopectin biosynthesis, quantitative expression profiles were constructed during three stages of seed development for two mutant lines and the parental wheat variety ‘C 306’ (Figs. 3 and 4). These two mutant lines contain about 7 % (‘TAC 6’) and 64 % (‘TAC 75’) amylose content. Of the 20 genes, 14 were starch biosynthesis genes [large and small subunits of ADP-glucose pyrophosphorylase (AGPase L and AGPase S), starch synthases including granule bound starch synthase (GBSSI) and four isoforms of soluble starch synthase (SSI, SSII, SSIII, and SSIV), three isoforms of starch branching enzymes (SBEI, SBEII, and SBEIII), and starch debranching enzymes including isoamylases and Pullulanase (ISA1, ISA2, ISA3, and PUL)]. Also found among the 20 genes were four starch degrading genes (Pho1, Pho2, AMY, and BMY) and two transcription factors (SPA and *TaRSR1*).

#### Expression pattern of starch metabolic genes in high amylose mutant line

The comparative quantitative gene expression analysis of 20 starch metabolic genes identified seven genes whose expressions were consistent throughout seed development in the high amylose mutant line (‘TAC 75’) in comparison with the parental wheat variety ‘C306’ (Fig. 3, Additional file 3). Of the seven, three genes (GBSSI, BMY, SPA) showed overexpression and four genes (SSIII, SBEI, SBEIII, ISA3) showed reduced expression during seed development in the high amylose mutant line. The expression of the remaining 13 starch metabolic genes was inconsistent, i.e. either high or low expression during seed development. In this study, overexpression of GBSSI in the high amylose mutant lines during the grain filling stage may lead to a higher accumulation of amylose as GBSSI plays a key role in the biosynthesis of amylose by elongating the linear α-1,4 glucan chain [38]. Over-expression of GBSSI enhanced amylose content in rice and wheat [36, 39] while silent or null mutants produced waxy or partial waxy wheats either lacking amylose or having low amounts of amylose [2, 23, 37, 40]. Overexpression of SPA may have a positive regulatory effect on amylose biosynthesis, given that the null mutant (osbzip58) for the rice homologue OsbZIP58 (a bZIP transcription factor) decreased amylose content in rice [41]. Amylose content can also be increased by the reduced expression or activity of the isoforms of SS, SBE, and isoamylases. Functional loss of SSIII in maize led to *dull-1* phenotype, which moderately increased the amylose content to 35–45 % [42]. Antisense inhibition of ISA in rice alters amylopectin structure [43]. SBEII is a key gene for amylopectin biosynthesis. Silencing of SBEII has enhanced amylose content [11, 13, 44]. Therefore, this study indicates that amylose accumulation in the high amylose mutant lines may have been the result of an overexpression of key genes for amylose biosynthesis as well as a downregulation of amylopectin and starch biosynthesis genes.

#### Expression pattern of starch metabolic genes in low amylose mutant line

The comparative quantitative gene expression analysis of 20 starch metabolic genes identified eight genes whose expressions were consistent throughout seed development in the low amylose mutant line ‘TAC 6’ in comparison to the parental wheat variety ‘C306’ (Fig. 4, Additional file 3). Of the eight genes, three (Pho2, AMY, and BMY) showed overexpression and five (AGPase L, GBSSI, SSI, SSIII, and *TaRSR1*) showed reduced expression during seed development in the low amylose mutant line. The expression of the remaining 12 starch metabolic genes was not consistent, i.e. either high or low expression during seed development. Amylases such as alpha and beta-amylases (AMY and BMY) along with starch phosphorylases, both plastidial (Pho1) and cytosolic (Pho2), play important roles in starch metabolism including hydrolysis and degradation [45]. They are starch modifying genes with major roles in maintaining starch structure and starch granule morphology. Silencing of starch phosphorylase in rice and potato showed alterations in starch structure [46, 47], whereas over expression of AMY and BMY affected the starch granules’ structure and baking quality [48]. Among the down-expressed genes, SSI is also considered a key gene for amylopectin biosynthesis. Its loss of function in rice and wheat increased amylose content and decreased amylopectin, with differences in the branching pattern [49]. Among highly expressed genes in the low amylose mutant line, SBEII is a key gene for amylopectin biosynthesis and its over expression increased amylopectin content in potato [17, 50]. Using co-expression analysis, a negative transcription factor, *RSR1* (rice starch regulator1), was identified in rice [20]. It is an APETALA2/ethylene-responsive element binding protein family transcription factor which significantly negatively regulates the expression of a few starch metabolic genes and thus modulates starch metabolism and starch-related phenotypes. In this study, the down expression of its wheat homologue, *TaRSR1*, in the low mutant line indicates that its effect may not have modulated starch metabolism. Therefore, this study indicates that amylopectin accumulation in the low amylose mutant line may have resulted from overexpression of key genes for amylopectin and starch biosynthesis as well as downregulation of amylose biosynthesis genes.

The differential gene expression analysis in the low and high amylose mutant lines in comparison to the parent variety support the involvement of other starch metabolic pathway genes such as phosphorylases, isoamylases, etc. in amylose/amylopectin biosynthesis in addition to the key biosynthesis genes (GBSS and SBE).

### Quantitative expression analysis of chromosome specific GBSSI alleles and SBEII isoforms

**Fig. 5:**
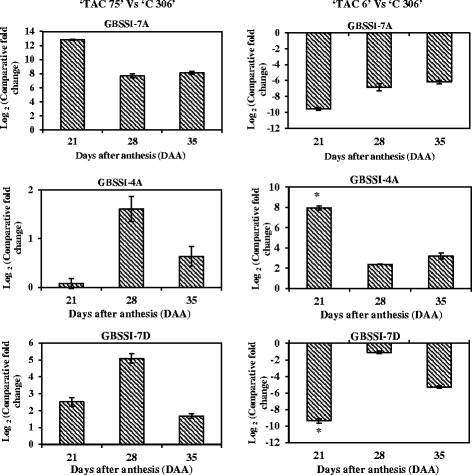
Real-time quantitative expression data (Log_2_ of fold change) of chromosome specific alleles of GBSSI during seed development of two mutant lines, ‘TAC 75’(amylose content - 64 %) and ‘TAC 6’(amylose content– 6.8 %), in comparison to the parent variety, ‘C 306’ (amylose content - 26 %). The seed development stages were 21, 28, and 35 days after anthesis (DAA). All the data are represented as mean ± SD from two biological and three technical replicates. The symbol ‘*’ indicates significant difference at *P* < 0.05

**Fig. 6:**
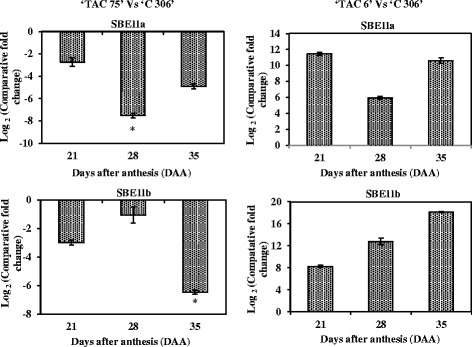
Real-time quantitative expression data (Log_2_ of fold change) of isoforms of SBEII during seed development of two mutant lines, ‘TAC 75’(amylose content - 64 %) and ‘TAC 6’(amylose content– 6.8 %), in comparison to the parent variety, ‘C 306’ (amylose content - 26 %). The seed development stages were 21, 28, and 35 days after anthesis (DAA). All the data are represented as mean ± SD from two biological and three technical replicates. The symbol ‘*’ indicates significant difference at *P* < 0.05

Quantitative expression analysis was performed to study the expression pattern of key genes of amylose (GBSSI’s homeologous alleles i.e. 7A, 4A, and 7D) and amylopectin (SBEII isoforms i.e. SBEIIa and SBEIIb) biosynthesis in the high and low amylose mutant lines in comparison to the parent variety ‘C 306’ (Figs. 5 and 6; Additional files 4 and 5). The GBSSI gene (or waxy protein) is responsible for amylose biosynthesis in storage tissues. Wheat endosperm contains three isoforms of the waxy protein encoded by the waxy (*wx*) loci. These loci are Wx-A1, Wx-B1, and Wx-D1, which are located on chromosomes, 7A, 4A (translocated from 7B), and 7D, respectively [2]. In comparison to the parent variety, the expression level of the three alleles of GBSSI (7A, 4A, and 7D) was high in the high amylose mutant line. Expression levels were low in ‘TAC 6’, except for the expression of the 4A allele during seed development (i.e. 21 to 35 DAA)(Fig. 5; Additional file 4). The down regulation of the 7A and 7D alleles indicates that the low mutant line may have null alleles of 7A and 7D (double-null) which may cause low amylose content (~7 %). Double-null partial waxy wheat with reduced amylose content was reported [51]. The loss of one, two, or three GBSSI isoforms results in the formation of single-null partial waxy, double-null partial waxy, and waxy wheat, respectively [4, 23, 52].

In comparison to the parent variety, the expression level of both SBEII isoforms (IIa and IIb) was low in the high amylose mutant line and, as expected, high in the low amylose mutant lines (Fig. 6; Additional file 5). Starch branching enzymes (SBEs) catalyze the hydrolysis of α-1,4 glycosidic linkages and re-attach the chain to α-1,6 positions and thus are involved in amylopectin biosynthesis. Two isoforms of SBEII have been reported in wheat and are classified as SBEIIa and SBEIIb [53]. Besides the higher expression of all three alleles of GBSSI in the high amylose mutant line, lower expression of both isoforms of SBEIIa and SBEIIb were also indirectly responsible for the elevation of amylose content. The suppression or null allele of both SBEIIa and SBEIIb have resulted in elevated amylose content in wheat [6, 7, 11, 13].

The expression patterns of GBSSI and SBEII indicate that loss of function of the two waxy alleles (GBSSI-7A and -7D) reduces the amylose content, while higher expression of both SBEII isoforms (SBEIIa and IIb) resulted in increased amylopectin content in the low amylose mutant line. In contrast, the expression patterns of GBSSI and SBEII indicate that overexpression of the three waxy alleles (GBSSI-7A, 4A, and 7D) elevates amylose content, while down expression of both SBEII isoforms (SBEIIa and IIb) decreases amylopectin in the high amylose mutant line.

## Conclusion

In this study a set of 101 EMS-induced mutant lines (M4 generation), showing variation in amylose and resistant starch content in seed, serve as useful germplasm or pre-breeding materials for genome-wide study and improvement of starch-based processing and nutritional quality in wheat. This population is also useful for the study of the genetic and molecular basis of amylose/resistant starch variation in wheat. Further, gene expression analysis of 20 starch metabolic genes in the two diverse mutant lines (low and high amylose mutant lines) indicates that in addition to key genes, several other genes (such as phosphorylases, isoamylases, and pullulanases) may also be involved in contributing to amylose/amylopectin biosynthesis.

## Methods

### Plant materials

The Indian hexaploid (2n = 6*x* = 42) bread wheat variety, ‘C 306’, was selected for developing EMS treated mutant lines for amylose/amylopectin variation. ‘C 306’ is popularly used in the production of chapatti (unleavened flat bread) [54]. The variety, ‘C 306’, used in this study was released in 1965 in India (pedigree: <RGN/CSK3//2*C591/3/C217/N14// C281>) and procured from Panjab Agricultural University, Ludhiana, India. Data for quantitative gene expression, amylose content, resistant starch content, and thousand-kernel weight was measured for three technical replicates of two biological replications each, both in the parent variety and the EMS derived mutant lines.

### Ethyl methanesulfonate (EMS) treatment and development of advanced generation of mutant lines

Approximately 5000 seeds, designated as M0 seeds, were soaked in 0.2 % EMS solution (survival rate of 60 %) for 16 h at room temperature (25–27 °C) with gentle agitation (50 rpm). The treated seeds were extensively washed with Milli-Q water and then kept in a fridge (4 °C) for 2 days before transferring to the NABI’s research farm. The treated seeds, referred to as M1 seeds, were sown individually. Individual spikes (M2 seeds) were selected from M1 plants and were sown the following season to grow M2 plants. Similarly, M3 and M4 seeds were collected as individual spikes to maintain homogeneity.

### Half-seed screening of mutant lines

Half-seed method was used for screening of amylose variation in about 1035 M3 mutant lines following the modified procedure of [32]. Briefly, seeds were cut horizontally in two parts containing an embryo and an endosperm. The half seed containing the endosperm was dipped in a different concentration of standard Iodine-Potassium Iodide solution [55]. The blue color intensity was recorded and its intensity was scored as colorless, intermediate, or dark colored. On the basis of color intensity, the lines were grouped into low, intermediate, and high amylose categories for further analysis.

### Measurement of amylose content, resistant starch content, and thousand kernel weight in mutant lines

For the measurement of amylose content (AC, %) and resistant starch content (RS, %) in grain starch of M3 mutant lines, starch granules were isolated following a modified protocol [56]. Amylose content in the extracted starch was measured using protocols described elsewhere [19]. AC was also reconfirmed using the modified Concanavaline A (Con A) precipitation method [57]. Con A is a lectin protein, which selectively binds with amylopectin and precipitates, leaving amylose in solution which can be used to determine the amount of amylose present. The percentage of amylose was calculated according to a standard curve prepared from pure potato amylose (Sigma-Aldrich, St. Louis, USA). The starch from High Amylose Maize (HAM) (Megazyme, Wicklow, Ireland) was used as a positive control for amylose estimation.

RS was measured following a modified procedure of Megazyme (Wicklow, Ireland) [58]. Briefly, ten milligrams of the extracted starch [10 mg, dry basis (db)] was dispersed in 1 ml DMSO and boiled at 100 °C for 30 min. The solubilized starch was partially hydrolysed to dextrins at 50 °C for 30 min with 300 U of α-amylase (Sigma-Aldrich, St. Louis, USA) and was completely hydrolysed into glucose with 30 U of amyloglucosidase (AMG) (Megazyme, Wicklow, Ireland) up to 120 min. The glucose content in both partially and completely hydrolysed starch samples was estimated following DNSA (3,5-dinitrosalicylic acid) method using a standard curve of the anhydrous D (+)-glucose (1 mg ml^−1^) (HiMedia, Mumbai, India) and absorbance was recorded at 540 nm [59].

### Quantitative gene expression analysis

Quantitative gene expression analysis of 20 starch metabolic pathway genes was performed during seed development in one low amylose mutant line (‘TAC 6’), one high amylose mutant line (‘TAC 75’), and the parent wheat variety ‘C 306’. The main spikes were harvested at 21, 28, and 35 days after anthesis (DAA), immediately frozen in liquid nitrogen, and stored at −80 °C for RNA extraction. The detailed protocols of RNA extraction, cDNA synthesis, and qRT-PCR are described elsewhere [60]. The gene and primer information of the 20 starch metabolic pathway genes and their isoforms were retrieved from Singh et al. [19]. Chromosome and isoform specific primer pairs of GBSSI (GBSSI- 7A, 4A, and 7D) and SBEII (SBEIIa and SBEIIb) were designed using Primer Express Software Tool version (3.0) (Table 2). Quantitative gene expression analysis was conducted in three technical replicates of two biological replications each, using a 7500 Fast Real-Time PCR System (Applied Biosystems, Forster City, CA, USA). Wheat ADP Ribosylation Factor (ARF) (AB050957.1) was used as an internal control gene for normalization of gene expression data and comparative fold change (Log_2_) was calculated following Livak and Schmittgen [61].

**Table 2:** Nucleotide sequences of primer pairs designed for chromosome specific alleles of GBSSI and isoforms of SBEII for quantitative real time PCR

Gene	NCBI ID	Forward primer (5’-3’)	Tm (°C)	Reverse primer (5’-3’)	Tm (°C)	Amplicon size (bp)
GBSSI-7A	EU719608.1	GAATGCGCTACGGAACGCCG	57.9	CCGCCTCAGCGTTGACTGCAA	58.3	109
GBSSI-4A	EU719610.1	TCGGCACGCCAGCCTACCAT	57.9	GGGTCATCGGCGAGGAGATT	55.9	143
GBSSI-7D	EU719612.1	GACAATAACCCCTACTTTTCTGGG	55.7	CAGGGCCGCAAAGGTGGCAT	57.9	139
SBEIIa	AF338432.1	GAACCGACTCAAGGCATTGTGG	56.7	CGGAGCCATCTTGACTACC	53.2	166
SBEIIb	AY740401.1	CAGTCGCCATCGCTGCTCTTC	58.3	ATGATCCCTGACGGCGGTAG	55.9	137

### Statistical analysis

Mean, standard deviation, linear regression, and Pearson’s correlation coefficient (r) were calculated using Microsoft Excel formulas. One-way analysis of variance (ANOVA) was used to study variation in amylose content, resistant starch content, and thousand-kernel weight in the replications for the mutant lines. Dunnett’s test, a *post hoc* test, was used to test the significant difference in the above traits in individual mutant lines with respect to the parent variety.

## Additional files

Additional file 1: Evaluation of 1035 (M3) mutant lines for amylose variation using five-time diluted standard Iodine-Potassium Iodide (I_2_–KI) solution. Time taken to develop blue color was recorded using a digital electronic stopwatch, while color intensity was scored as zero for no color development, + for light blue color, and ++ for deep blue color. All the data was given as a mean of three seeds. (XLSX 58 kb)
Additional file 2: Measurement of amylose content variation in the 101 (M4) mutant lines by traditional colorimetric method using five-time diluted standard Iodine-Potassium Iodide (I_2_–KI) solution. The data was taken in three technical replications and each represented as mean ± SD. (DOC 56 kb)
Additional file 3: Normalized threshold cycle (ΔCт) of 20 starch metabolic genes during seed development. The data was taken at three stages of seed development (21, 28, and 35 days after anthesis, DAA) in two mutant lines, ‘TAC 75’(amylose content - 64 %) and ‘TAC 6’(amylose content– 6.8 %), in comparison to the parent variety, ‘C 306’ (amylose content - 26 %). The data was taken in two biological replications, each represented as mean ± SD from three technical replicates. Wheat *ADP Ribosylation Factor (ARF*) was used as an internal control gene for normalization. (XLSX 23 kb)
Additional file 4: Normalized threshold cycle (ΔCт) of chromosome specific alleles of GBSSI (GBSSI–7A, GBSSI–4A, and GBSSI–7D) during seed development. The data was taken at three stages of seed development (21, 28, and 35 days after anthesis, DAA) in two mutant lines, ‘TAC 75’(amylose content - 64 %) and ‘TAC 6’(amylose content– 6.8 %), in comparison to the parent variety, ‘C 306’ (amylose content - 26 %). The data was taken in two biological replications, each represented as mean ± SD from three technical replicates. Wheat *ADP Ribosylation Factor (ARF*) was used as an internal control gene for normalization. (XLSX 12 kb)
Additional file 5: Normalized threshold cycle (ΔCт) of two SBEII isoforms (SBEIIa and SBEIIb) during seed development. The data was taken at three stages of seed development (21, 28, and 35 days after anthesis, DAA) in two mutant lines, ‘TAC 75’(amylose content - 64 %) and ‘TAC 6’(amylose content– 6.8 %), in comparison to the parent variety, ‘C 306’ (amylose content - 26 %). The data was taken in two biological replications, each represented as mean ± SD from three technical replicates. Wheat *ADP Ribosylation Factor (ARF*) was used as an internal control gene for normalization. (XLSX 10 kb)

## Abbreviations

Con A: Concanavaline A
CTC: Concentration-time-color intensity
DAA: Days after anthesis
EMS: Ethyl methanesulfonate
HAM: High amylose maize
KL: Kernel length
KW: Kernel weight
PG: Phytoglycogen
qRT-PCR: Quantitative real-time polymerase chain reaction
RS: Resistant starch
TILLING: Targeting induced local lesions IN genomes
TKW: 1000-kernel weight
wx: Waxy
