# Supplementary material for: Development of EMS-induced mutation population for amylose and resistant starch variation in bread wheat (Triticum aestivum) and identification of candidate genes responsible for amylose variation
Source: BMC Plant Biol. 2016 Oct 6;16:217. doi: 10.1186/s12870-016-0896-z (PMC5054548; doi:10.1186/s12870-016-0896-z)
Supplement: Additional file 2: — Measurement of amylose content variation in the 101 (M4) mutant lines by traditional colorimetric method using five-time diluted standard Iodine-Potassium Iodide (I2–KI) solution. The data was taken in three technical replications and each represented as mean ± SD. (DOC 56 kb) [file 12870_2016_896_MOESM2_ESM.doc]

**Table S1. Amylose content measured on the 101 (M4) mutant lines using traditional I_2_-KI method.**

| **Mutant lines** | **Amylose (%)** | **Mutant lines** | **Amylose (%)** |
| --- | --- | --- | --- |
| ‘C 306’ (parent) | 26.0 ± 0.4 | TAC 708 | 25.2 ± 0.2 |
| ‘High amylose maize’ (66%) standard | 66.0 ± 0.4 | TAC 711 | 18.2 ± 0.2 |
| TAC 6 | 06.3 ± 0.0 | TAC 713 | 13.4 ± 0.0 |
| TAC 28 | 73.9 ± 0.4 | TAC 730 | 12.5 ± 0.3 |
| TAC 35 | 67.5 ± 0.4 | TAC 737 | 13.9 ± 0.5 |
| TAC 51 | 68.7 ± 0.2 | TAC 741 | 16.5 ± 0.4 |
| TAC 71 | 68.9± 0.1 | TAC 747 | 24.5 ± 0.3 |
| TAC 74 | 69.2 ± 0.5 | TAC 748 | 24.9 ± 0.4 |
| TAC 75 | 66.9 ± 0.4 | TAC 765 | 15.9 ± 0.3 |
| TAC 104 | 03.4 ± 0.6 | TAC 766 | 19.3 ± 0.3 |
| TAC 137 | 17.9 ± 0.3 | TAC 781 | 29.4 ± 0.0 |
| TAC 163 | 16.3 ± 0.5 | TAC 790 | 42.1 ± 0.5 |
| TAC 176 | 14.3 ± 0.2 | TAC 810 | 19.1 ± 0.0 |
| TAC 197 | 25.2 ± 0.3 | TAC 824 | 49.9 ± 0.4 |
| TAC 237 | 07.0 ± 0.3 | TAC 831 | 33.2 ± 0.2 |
| TAC 243 | 41.0 ± 0.3 | TAC 846 | 08.5 ± 0.2 |
| TAC 273 | 25.2 ± 0.1 | TAC 869 | 26.9 ± 0.2 |
| TAC 287 | 34.5 ± 0.2 | TAC 880 | 12.4 ± 0.2 |
| TAC 288 | 10.5 ± 0.5 | TAC 902 | 20.4 ± 0.2 |
| TAC 308 | 37.1 ± 0.3 | TAC 903 | 31.7 ± 0.2 |
| TAC 354 | 21.1 ± 0.7 | TAC 914 | 17.5 ± 0.3 |
| TAC 358 | 02.9 ± 0.5 | TAC 917 | 27.1 ± 0.3 |
| TAC 360 | 47.0 ± 0.2 | TAC 942 | 33.8 ± 0.1 |
| TAC 362 | 33.6 ± 0.6 | TAC 947 | 50.8 ± 0.4 |
| TAC 369 | 40.7 ± 0.5 | TAC 955 | 26.5 ± 0.2 |
| TAC 374 | 13.8 ± 0.6 | TAC 975 | 55.1 ± 0.2 |
| TAC 380 | 25.9 ± 0.3 | TAC 981 | 12.5 ± 0.3 |
| TAC 381 | 29.2 ± 0.2 | TAC 989 | 32.0 ± 0.5 |
| TAC 399 | 75.7 ± 0.4 | TAC 990 | 26.8 ± 0.1 |
| TAC 404 | 46.1 ± 0.5 | TAC 1024 | 50.8 ± 0.3 |
| TAC 418 | 35.3 ± 0.5 | TAC 1025 | 11.6 ± 0.5 |
| TAC 419 | 32.0 ± 0.3 | TAC 1026 | 12.6 ± 0.2 |
| TAC 421 | 36.4 ± 0.2 | TAC 1046 | 16.1 ± 0.4 |
| TAC 423 | 20.0 ± 0.2 | TAC 1054 | 14.6 ± 0.3 |
| TAC 428 | 43.4 ± 0.2 | TAC 1068 | 35.9 ± 0.2 |
| TAC 437 | 51.5 ± 0.3 | TAC 1072 | 37.3 ± 0.1 |
| TAC 457 | 34.9 ± 0.2 | TAC 1075 | 22.4 ± 0.2 |
| TAC 477 | 35.5 ± 0.3 | TAC 1081 | 12.8 ± 0.2 |
| TAC 536 | 16.4 ± 0.1 | TAC 1090 | 57.1 ± 0.2 |
| TAC 539 | 16.1 ± 0.2 | TAC 1151 | 53.5 ± 0.5 |
| TAC 560 | 19.7 ± 0.0 | TAC 1168 | 07.1 ± 0.0 |
| TAC 587 | 13.1 ± 0.3 | TAC 1171 | 63.4 ± 0.6 |
| TAC 606 | 6.8 ± 0.0 | TAC 1193 | 73.8 ± 0.4 |
| TAC 622 | 42.7 ± 0.2 | TAC 1194 | 69.5 ± 0.4 |
| TAC 623 | 20.9 ± 0.4 | TAC 1201 | 69.0 ± 0.2 |
| TAC 636 | 42.0 ± 0.2 | TAC 1202 | 68.7 ± 0.1 |
| TAC 662 | 44.4 ± 0.5 | TAC 1207 | 35.7 ± 0.2 |
| TAC 681 | 26.9 ± 0.7 | TAC 364 | 40.2 ± 0.5 |
| TAC 696 | 46.8 ± 0.3 | TAC 172 | 31.5 ± 0.3 |
| TAC 703 | 48.2 ± 0.2 | TAC 988 | 23.8 ± 0.2 |
| TAC 14 | 62.8 ± 0.6 | TAC 1105 | 25.8 ± 1.0 |
